# Application of Distributed Probability Model in Sports Based on Deep Learning: Deep Belief Network (DL-DBN) Algorithm for Human Behaviour Analysis

**DOI:** 10.1155/2022/7988844

**Published:** 2022-02-18

**Authors:** Tianyang Liu, Qizhe Zheng, Ling Tian

**Affiliations:** ^1^Physical Education Department, Guangzhou Sport University, Guangzhou 510500, Guangdong, China; ^2^Computer Research Laboratory, South China University of Technology, Guangzhou 510641, Guangdong, China; ^3^Public Physical Education Department, Guangzhou Maritime Institute, Guangzhou 510725, Guangdong, China

## Abstract

With the increased development of information technology, almost all the sectors have been developed. Age, educational qualifications, gender, and other factors have no bearing on acquiring knowledge in information technology.Most humans use mobile phones and other gadgets to make their lives easier. Machine Learning techniques are used to analyse the given data and aid in the classification or prediction of the dataset depending on the problem statement. It is significant to determine human behaviour analysis in the context of sports. In this research, the Deep Learning-Deep Belief Network (DL-DBN) algorithm is implemented with probability to analyse human behaviour in sports and implement a distributed probability model for classifying the behavior. The classification results have shown that the accuracy for strength training is both the maximum and the smallest, reaching 99% and 71%, respectively.

## 1. Introduction

Due to the growth of China's sports programs, the sports audience in China varies with respect to behavior, social class, and urban type. Significantly, the extraction of human activity knowledge from huge video data sources has become a pressing issue in a variety of fields. If intelligent video surveillance is used, video can also be instantly designed and analysed [1]. Human behaviours can also be accurately recognised in the real world and can generate security warnings with time settings. They are expanding the range of applications available in public spaces like transportation hubs, airports, and public transport. As a result, human behaviour recognition does have theoretical and managerial contributions, and it has become a focus of research in many fields. Body actions range from the primary body movements to the much more complex joint activities of the human body, such as leg movements during sports. Body action recognition is frequently studied from both practical and theoretical perspectives. In theory and research, activity recognition involves data acquisition and processing. Previously, some wearable technologies were used to collect data on body movement. Even though the obtained action data was rich, it had significant ineffectiveness, expense, and environmental impact flaws. Body action data can be gathered thanks to the continuous updating and modifying of video capture devices. In computer vision, deep learning is widely used in image processing, classification, analysis, and prescriptive analytics. The study's goal is to mechanically collect information on human supporting events and behavior-related video databases in order to provide precise recognition and analysis of physical movements [2]. The evaluation of multi-scale digital data, the advancement of spatiotemporal Deep Belief Network (DBN), and the utterly various pooling methods are thought of as the focal points that aid in enhancing the networks in the Deep Learning (DL) mechanism. Furthermore, an individual's sports behaviour complete representation is designed to support specific spatiotemporal options. Crowd intelligence mechanism aids in reviewing and players' future results are predicted and they are fingered in a National Basketball Association league All-Star game [3]. The goal of the study is to analyse the soccer player's behaviour using machine learning algorithms with various classifying parameters that are derived from OPTA's (Optimum Performance Theoretically Attainable) on-ball event [4]. The focus of encounter analysis shifts indefinitely and includes psychological factors such as visual data, perceived notions, and process. In highly dynamic tasks, such as sports decision-making, these factors become even more important in laying the groundwork for diagnostic methods and reconciling learning environments. Despite the fact that most recent research focuses on behavioural options, with analysis of complex eye trailing knowledge, which is more complex than any other behavioural mechanism, sociallife can be a tricky balancing act [5]. To graciously coordinate with someone's partners, one should anticipate their actions. In this section, we look at how people predict the same actions of others [6]. To determine whether or not individuals have accurate information about the chances of transitioning between actions, datasets were used to make an analysis using the rating system and the correlating behaviour of the players. The researcher correlates expression once more for odds of victory in a game in a real match under the assumption that the outcome of each goal is identically and collectively distributed [7]. The necessary properties of the equation square measure have been evaluated and illustrated. The accuracy of the formula is tested by comparing determined proportions to expected values using information from the 2007 Suburban Grass Tennis Championship games. We have a tendency to additionally derive expressions for the possibility of many alternate solution milestones in a match, such as winning an overtime, winning a collection, having won a match, and being tired from an event of service all the way down to winning a selection. The researcher recommends a sports-flesh-supported computer vision start-chasing technology analytical framework [8]. Visual target pursuit is a critical analysis field of computer vision and motion mechanical phenomena, and it will provide not only the goal, but also the initial data movement assessment, scene understanding, behaviour or activity recognition in intellectual police work, human-computer interaction, golem graphic navigation, and movement recognition. The supported field has a wide range of application possibilities. Despite the increasing interest in quantifying and modeling grading dynamics in skilled sporting events, little is thought about what patterns or principles, if any, cut across completely different sports are discussed in [9]. The analysis of communicating directly in soccer allows the spatial distribution of players throughout matches to be delineated, which improves understanding of strategic restrictions on player behavioural dynamics. The objective of study would have been to identify players' spatial constraints that affect the exploratory plan of action behaviour and impact the perception space of players in control of the ball, likewise inter-player passing conversations [10]. The most important analysis is the rapid and dynamical analysis of advanced technology to recognise human activity automatically [11]. A live camera is implemented to monitor the health in order to determine whether or not the operation was successful. computer vision space to find out an individual's activity with the help of object detection, feature extraction, untidy professional experience, occlusion, and trying to apply deep learning approaches to find success in the answer. In order to conserve experimental rigour, researchers learning accommodating behaviour in body movement systems have historically used simplified, laboratory-based movement models [12]. Brunswikian science raises concerns about the representativeness of many of the widely used model systems for learning, despite the fact that movements are organised around activities, objects, and surfaces of dynamic environments. The study's goal was to assess the current research on injury interference methods for elite athletes who compete in major international sports competitions, as well as to look at the major core strategic factors [13]. By preparing for international competitions through an in-depth literary evaluation and discussion of injuries and diseases, the inspiration for the injury interference strategy for elite athletes preparing for international competitions was identified. Collective behaviour is frequently defined as humans' ability to integrate with others in a fancy environment [14]. Sports provide excellent examples of this continuous interplay, requiring higher cognitive processes and alternative sensory processing to regulate individual selections to team self-organization and vice versa. When considering team members as periodic segment oscillators, space-time relation analyses are frequently used to model teamwork [15]. Coaching behaviour is compared with common behavior, losses are calculated, and coaching procedures are scientifically analysed. Coaches must verify and make sure that each action taken by the football players meets minimum requirements. Another of the world's main research topics is the use of AI technology analysis to investigate human behaviour [16]. As a DL algorithm, DBN does have advantages in human activity recognition and representation. This can process a variety of input data as well as extract the interconnection between adjoining times. The sources of the data for actions are then provided without presuming the distribution of action features. As a result, it can be used in action recognition. As a result, DL-DBN is improved, and a complete representation of human sporting events related to specific temporal and spatial features is proposed. Massive amounts of video data must be extracted, recognised, and analysed to extract, recognise, and analyse human sporting event behaviour. The developed algorithm has been tested on UCF101 datasets, laying the groundwork for future sports development and muscular identification through China.

## 2. Materials and Methods

By using advanced technology in sports, anyone can automatically receive data on human sports behaviour from a large volume of video data with delivery acknowledgment as well as analysis of physical movements. Even though scientific and technological advances allow fast data transmission on volumes of data to explore by extracting information about human behavior, large video data sets represent an immediate problem in various fields. The video could be automatically modelled and analysed if intelligent surveillance cameras are used. Human behaviours could be recognised in real-time by increasing the accuracy and timing of security warnings. As a result, human behaviour recognition does have practical and theoretical implications, and it has become a focus of research in so many fields. When images could indeed recognise image frames or sequential, human activity recognition has become a classification problem. In this study, a distributed probability model has been implemented and the data analysed using a deep learning approach.

**Step 1 .**

Multi-category classification, on the other hand, is much more frequent. For this, there seem to be typically two options:

*n* Binary classification methods but also multi-classifier soft max structural equation modeling spread to logistic regression. Whether there is currently a multi-category classification task conveyed as *s*(*i*)1,2,…, *n*, with such a total of *n* categories. Equation (1) shows the categorization probability presumed in soft max correlation classification for the test data *c*.

(1) $$\begin{array}{c} E_{\theta }\left(c^{\left(i\right)}\right)=\left[\begin{array}{c} F\left(s^{\left(i\right)}=1|c^{\left(i\right)};\theta \right)\\ F\left(s^{\left(i\right)}=2|c^{\left(i\right)};\theta \right)\\ \ldots \\ F\left(s^{\left(i\right)}=n|c^{\left(i\right)};\theta \right) \end{array}\right]\\ =\frac{1}{\sum_{j=1}^{n}e^{\theta_{j}^{L}s^{\left(i\right)}}}\left[\begin{array}{c} e^{\theta_{1}^{L}c^{\left(i\right)}}\\ e^{\theta_{2}^{L}c^{\left(i\right)}}\\ \ldots \\ e^{\theta_{n}^{L}c^{\left(i\right)}} \end{array}\right]. \end{array}$$

**Step 2 .**

*E*_*θ*_(*c*^(*i*)^) denotes the parameters of the model, which are *e*^*θ*_*j*_^*L*^*s*^(*i*)^^ represented by a *n*-line structure. As shown in equation (2), *F*(*s*^(*i*)^=1,2,…, *n|c*^(*i*)^; *θ*) each boundary could be viewed as an individual categories classifier attribute.

(2) $$\begin{array}{c} \theta =\left[\begin{array}{c} \theta_{1}^{L}\\ \theta_{2}^{L}\\ \vdots \\ \theta_{n}^{L} \end{array}\right]. \end{array}$$

**Step 3 .**

1/∑_*j*=1_^*n*^*e*^*θ*_*j*_^*L*^*s*^(*i*)^^ normalizes the probabilistic model so that the *θ*_1_^*L*^ total probability is the one and the decision variables for the system is shown in equation (3).

(3) $$\begin{array}{c} D\left(\theta \right)=-\frac{1}{p}\left[\sum_{i=1}^{p}\sum_{j=1}^{n}1\left\{s^{\left(j\right)}=j\right\}\right]\log\frac{\theta_{j}^{L}c^{\left(i\right)}}{\sum_{j=1}^{n}e^{\theta_{j}^{L}c^{\left(i\right)}}}. \end{array}$$

**Step 4 .**

The valuation rules for such suggestive function *D*(*θ*) are log*θ*_*j*_^*L*^*c*^(*i*)^/∑_*j*=1_^*n*^*e*^*θ*_*j*_^*L*^*c*^(*i*)^^. The scenarios of a *n*/*F*(*s*^(*i*)^=*j|x*^(*i*)^; *θ*) categories are then accumulated by Soft max correlation. equation (4) calculates the likelihood that *c* is classified into *j* classifications.

(4) $$\begin{array}{c} \log\;F\left(s^{\left(i\right)}=j|x^{\left(i\right)};\theta \right)=\frac{\theta_{j}^{L}c^{\left(i\right)}}{\sum_{j=1}^{n}e^{\theta_{j}^{L}c^{\left(i\right)}}}. \end{array}$$

**Step 5 .**

The decision variables generalization of regression models is shown in equation (3). The correlation objective functions are depicted in equation (5).

(5) $$\begin{array}{c} D\left(\theta \right)=-\frac{1}{p}\left[\sum_{i=1}^{p}\sum_{j=1}^{n}1\left\{s^{\left(i\right)}=j\right\}\log\;F\left(s^{\left(i\right)}=j|c^{\left(i\right)};\theta \right.\right]. \end{array}$$

**Step 6 .**

Correspondingly, ∇_*θ*_*j*__*D*(*θ*) a recursive optimization technique, including (1{*s*^(*i*)^=*j*} − *F*(*s*^(*i*)^)=*j|c*^(*i*)^; *θ*) the numerical solution, can minimize the objective functions in this equation. As a result, equation (6) shows how to compute the derivative of the function of an error function.

(6) $$\begin{array}{c} \nabla_{\theta_{j}}D\left(\theta \right)=-\frac{1}{p}\sum_{i=1}^{p}\left[c^{\left(i\right)}\left(1\left\{s^{\left(i\right)}=j\right\}-F\left(s^{\left(i\right)}\right)=j|c^{\left(i\right)};\theta \right)\right]. \end{array}$$

**Step 7 .**

In equation (6), ∇_*θ*_*j*__*D*(*θ*) is a vector, and its *l*^th^(∂*J*(*θ*)/∂*θ*_*jl*_) is the *l*^th^ be either in the price function's *j*^th^ category. Above that the equation is fed into the gradient descent as well as revised recursively to minimize the objective function. The *e*^(*θ*_*l*_ − *ψ*)_*j*_^*L*^*c*^(*i*)^^ failure function's significance does not really change when the same number is deducted out of each solution obtained parameter, implying that ∑_*l*=1_^*n*^*e*^*θ*_*j*_^*L*^*c*^(*i*)^^ the parameter may not be the only solution. The evidence procedure is depicted in equation (7).

(7) $$\begin{array}{c} F\left(s^{\left(i\right)}=j|c^{\left(i\right)};\theta \right)=\frac{e^{{\left(\theta_{l}-\psi \right)}_{j}^{L}c^{\left(i\right)}}}{\sum_{l=1}^{n}e^{{\left(\theta_{l}-\psi \right)}_{j}^{L}c^{\left(i\right)}}}=\frac{e^{\theta_{j}^{L}c^{\left(i\right)}-e^{-\psi_{j}^{L}c^{\left(i\right)}}}}{\sum_{l=1}^{n}e^{\theta_{j}^{L}c^{\psi }}e^{-\psi_{j}^{L}x^{\left(i\right)}}}=\frac{e^{\theta_{j}^{L}c^{\left(i\right)}}}{\sum_{l=1}^{n}e^{\theta_{j}^{L}c^{\left(i\right)}}}. \end{array}$$

**Step 8 .**

To persecute increased model *θ*_*ij*_^2^ parameters and make sure that functional form is the stringent function defined, *p*, *n* load energy loss is added to it. equation (8) depicts the cost function as it approaches the efficient optimization solution.

(8) $$\begin{array}{c} D\left(\theta \right)=-\frac{1}{p}\left[\sum_{i=1}^{p}\sum_{j=1}^{n}1\left\{s^{\left(i\right)}=j\right\}\log\frac{e^{\theta_{j}^{L}c^{\left(i\right)}}}{\sum_{l=1}^{n}e^{\theta_{j}^{L}c^{\left(i\right)}}}\right]+\frac{\lambda }{2}\sum_{i=1}^{n}\sum_{j=1}^{t}\theta_{ij}^{2}. \end{array}$$

**Step 9 .**

In equation (8), *λ* > 0. equation (9) illustrations the fractional derived function.

(9) $$\begin{array}{c} \nabla_{\theta_{j}}D\left(\theta \right)=-\frac{1}{p}\sum_{j=1}^{p}\left[c^{\left(i\right)}\left(1\left\{s^{\left(i\right)}=j\right\}-F\left(s^{\left(i\right)}=j|c^{\left(i\right)};\theta \right)\right)\right]+\lambda \theta_{j}. \end{array}$$

**Step 10 .**

Finally, by minimizing the ∇_*θ*_*j*__*D*(*θ*) objective function, an utilizable soft max correlation {*s*^(*i*)^=*j*} − *F*(*s*^(*i*)^=*j|c*^(*i*)^; *θ*) classification model can be obtained as in equation (10).

(10) $$\begin{array}{c} \nabla_{\theta_{j}}D\left(\theta \right)=-\frac{1}{p}\sum_{j=1}^{p}\left[c^{\left(i\right)}\left(1\left\{s^{\left(i\right)}=j\right\}-F\left(s^{\left(i\right)}=j|c^{\left(i\right)};\theta \right)\right)\right]+\sum_{j=1}^{n}1\left\{s^{\left(i\right)}=j\right\}\log\frac{e^{\theta_{j}^{L}c^{\left(i\right)}}}{\sum_{l=1}^{n}e^{\theta_{j}^{L}c^{\left(i\right)}}}. \end{array}$$

Eventually, by minimizing a cost function, a (−*D*(*c*, *s*, *φ*) utilizable nonlinear activation regression classification model can be obtained as in equation (11) the probability is one decision variable equation for the system.

(11) $$\begin{array}{c} F\left(c,s|\theta \right)=\frac{1}{s\left(\varphi \right)}\text{exp}\left(-D\left(c,s,\varphi \right)\right.. \end{array}$$

The probability of ∫_*e*′∈*s*,*h*′∈*H*_) categorization presumed in correlation classification is calculated as in equation (12).

(12) $$\begin{array}{c} S\left(\theta \right)=\int_{e^{\prime }\in s,h^{\prime }\in H}\text{exp}\left(-D\left(c,s,\theta \right)\right), \end{array}$$

(13) $$\begin{array}{c} F\left(s|\theta \right)=\frac{\text{exp}\left(-F\left(z,\theta \right)\right)}{\int_{S^{\prime }\in S}\text{exp}\left(-F\left(s,\theta \right)\right)}. \end{array}$$

Scoring Goals Probability Model for *F*(*s*, *φ*) Scenes Using Simultaneous Positional Information as in equation (14).

(14) $$\begin{array}{c} F\left(s,\theta \right)=-\int_{h\in c}\exp\left(-F\left(s,c,\theta \right)\right). \end{array}$$

The probability value *θ* is classified into *i*, *j* categories in most cases, maximum probability convolution is used in the deep network, which is activated only after at least many of its corresponding invisible deep network is activated as in equation (15).

(15) $$\begin{array}{c} \left(\theta \right)=-\frac{1}{p}\left[\sum_{i=1}^{p}\sum_{j=1}^{n}1\left\{s^{\left(i\right)}=j\right\}\log\frac{e^{\theta_{j}^{L}c^{\left(i\right)}}}{\sum_{l=1}^{n}e^{\theta_{j}^{L}c^{\left(i\right)}}}\right]. \end{array}$$

The probabilities of the hidden units are acquired by the probability integral's uniformly distributed in each layer, as shown by equations (15) and (16).

(16) $$\begin{array}{c} F\left(sc_{a}^{\delta }=-F_{a}^{\delta }|c!\right)=1-F\left(F_{a}^{\delta }|h!\right). \end{array}$$

The probability density function is constructed in such a way that the sum among all probabilities is on equation (17).

(17) $$\begin{array}{c} F\left(sc_{a}^{\delta }=h_{a,s}^{\delta }|h!\right)=\frac{1}{\left|S_{a}^{\delta }\right|}F\left(F_{a}^{\delta }|h!\right). \end{array}$$

**Figure 1:**
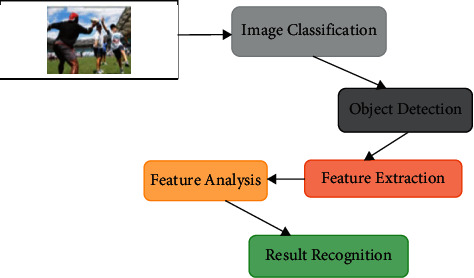
Overall Architecture for human behaviour of sports.

In Figure 1, activities may produce similar estimates in human behaviour processing and facial expression due to viewpoint changes. Environmental factors, such as lighting changes and consensual covering, make recognising human behaviour difficult. As the principal components of human behaviour recognition, selecting features as well as efficient characteristics from video image sequence data to define the hand movement's condition can decrease spatiotemporal offering various levels of calculation sophistication. Choosing suitable features characterises sports behaviours, and a classification model is trained to classify human actions using DL methods, yielding the final recognition performance.

## 3. Results and Discussion

The study employed distributed probability model for analyzing the human behavior of sports. The data has been validated by using deep learning approach.

In Table 1, it is observed that the UCF101 has a total of 101 action classes that we have categorised into 5 categories: Human-Object Communication, Body-Motion Then, effectively delegate conversation, playing ensemble equipment, and sports. UCF101 is an expansion of UCF100, which included 100 action classes such as baseball pitching, basketball shooting, bench pressing, and so on.

**Figure 2:**
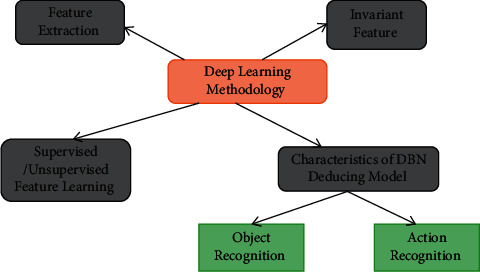
The DL community's agreement on extracting features and continuing to learn in DBN.

Fully automated learning to acquire robustness in image features representation in huge unsupervised learning (including pictures and videos) has become a critical task for the next generation of advanced vision techniques. Unsupervised feature learning is receiving a huge amount of attention in object recognition. In the DL application, investigators in object recognition and cognitive science have also reached the general agreement shown in Figure 2 in the extracting of features and unsupervised feature extraction.

In Table 2, it is observed that feature extraction (9%), slides playing (15320), human-object interaction with feature learning (dynamic), resource (TV, movies, YouTube), and time duration (1200 mins) are the action types with the object with action recognition (8.67 sec). Sports activities achieve high precision because they usually require distinguishable motions, making classification easier. Furthermore, when compared with other types of action, the backstory in sports clips is usually less cluttered. Human social interaction clips, unlike sporting events, generally have a crowded experience. Furthermore, the informative motions limit a tiny fraction of the movements in the frames, which explains why this ad has low recognition performance.

**Figure 3:**
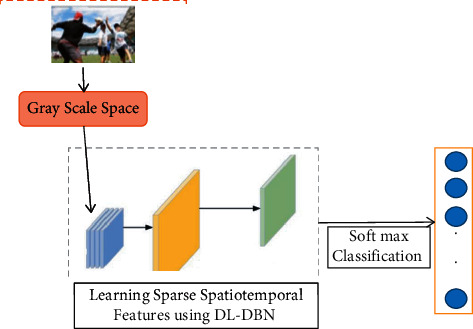
Model for recognising human sports behaviour based on sparsity spatial and temporal features.

In Figure 3, multi-resolution representations are used as DL-different DBN's connection attributes to collectively learn multi-scale attributes as well as the information interaction of different scales if learning the sparse spatio-temporal features of human sports behaviour.

In Table 3, the evolution of supervised and unsupervised feature extraction to the position of the frame, on the other hand, is more important than in the spatial dimension. The multi-scale DL-DBN, in particular, uses various scale representations of DL-input DBN's videos to classify the values of various channels to collectively learn spatio-temporal features from multiple scales.

**Figure 4:**
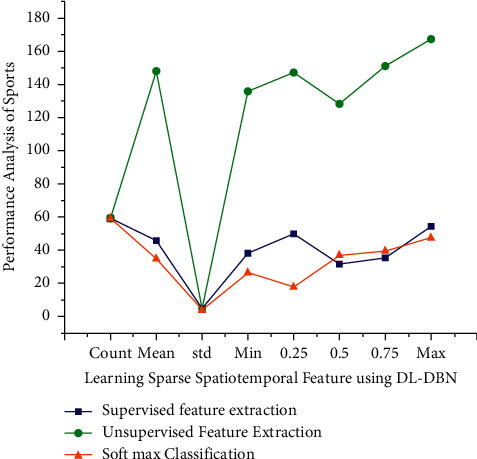
Performance analysis for sports spatiotemporal feature using DL-DBN.

In Figure 4, the enhanced DL-DBN model is pre-trained using greedy soft max classification. Also, every model's input strands are trained at random, beginning with its sub layer. The supervised learning expression is then reconfigured and input to another layer. This process continues indefinitely all through the training till all the layers have been trained. After that, the entire infrastructure has been trained. The (un)supervised expressions of each layer in the video could be extracted.

In Table 4, it is observed that performance analysis for sports spatiotemporal features using DL-DBN, a method of video preprocessing, object recognition, feature extraction, as well as classification, is used by DL applications in action recognition. Furthermore, the spatio-temporal feature sensor selects a general-interest region, limiting the amount of data that must be recognised significantly. Following the extraction of the pertinent points, the feature characteristics employ the DL model and reject all location information.

**Figure 5:**
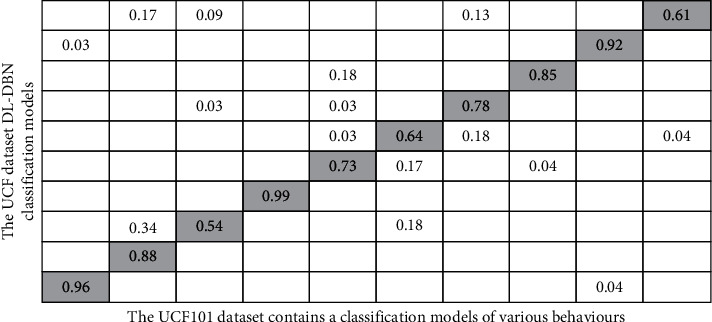
The UCF101 dataset contains a classification models of various behaviours.

Figure 5 represent UCF101 dataset contains a classification models advancement of DL techniques. It reveals that human activity recognition has emerged as a data analysis access point in computer vision and image processing, attracting considerable interest from academics across disciplines. In this paper, we suggest a human sports behaviour recognition model with certain spatial and temporal attributes by enhancing DBN. In this classification, the matrix calculation at the diagonal is classified into various behaviours. In the diagonals below and above, human activity recognition has emerged in a data analysis that represents the access point in the images. Moreover, based on this analysis of human activity recognition in various sports behaviors, In Table 5, the classification and recognition regression model of ten behaviours in UCF was created by analyzing the accuracy in the UCF sports dataset, indicating that the suggested technique has sensible accuracy rates. Furthermore, the prediction accuracy for strength training is the maximum and the smallest, reaching 99% and 71%, respectively. Similar attitudes, including such stomping and having to run, are misclassified.

**Figure 6:**
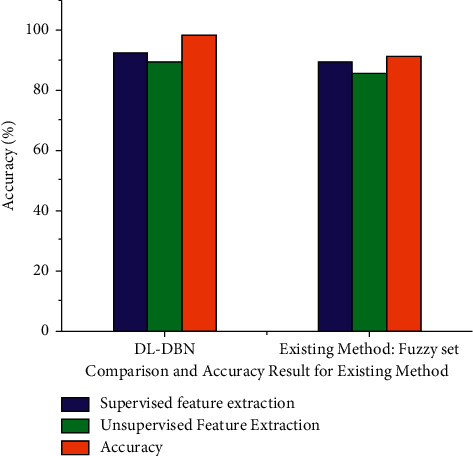
Comparison and overall accuracy result analysis for existing system.

The overall comparison result analysis for the existing system based on the supervised and unsupervised feature extraction and calculated for the accuracy. The DL-DBN method supervised feature extraction (92,45%), unsupervised feature extraction (89.56%) and the overall accuracy (98.56%). The existing method supervised feature extraction (89.34%), unsupervised feature extraction (85.56%) and overall accuracy (91.42%) it compared the existing method is best for in our proposed method and it provide for the exact result (Figure 6).

As a result, this model is enhanced further by modeling separately in time and space, making it more invariant in spatio-temporal transformation. A hierarchical approach is used the dispersed probability model, which gets to know temporal and spatial features extraction from videos using reinforcement learning, is used here. Specifically, as the introduction module, the convolution restricted machine tries to learn a hierarchical structure of the original data structure. From of the high to low, its structure becomes increasingly complicated, and the temporal and spatial Deep Convolution Network is named after the steady rise in representations.

A meta-heuristic based hierarchical pre-training algorithm is used to train the improved deep learning DBN model. Also every model's input layers are educated at random beginning with its lowest layer. The hidden nodes representation probability is then rearranged and insight to another layer. This process repeats indefinitely all throughout training until all layers have been trained. After the entire network has been trained, the hidden terminal probability expressions of any given layer with in video could be extracted.

## 4. Conclusion

With the development of science and technology, so do the desires for the current levels of the sport competitive rivalry. Having recent rapid development of the international sports, numerous social issues in sports had also begun to surface. Our country's rapid development in this field has resulted in major accomplishments as well as numerous problems, such as inordinate participation of athletes in self - employed, unhealthful dietary habits and weight control, and ability to participate in employment once injured. Typically, this is a demonstration of the issue of positive variance behaviour. Deep learning algorithms are being used in a variety of fields, including UAV (Unmanned Aerial Vehicle) driving, visual acknowledgment, target pursuit, behaviour recognition, and others. Several scientists recommend the analysis of aim pursuit and change based on supported deep learning models for athletes' mechanical occurrence and behaviour capture in the sports world. This research focused on the human behavior in sports with the aid of Deep Learning technology and probability statistics is implemented and the results were discussed. A novel Deep Learning-Deep belief network (DL-DBN) algorithm is proposed to make the analysis of the human behavior classification. The proposed model is observed to provide improved results with an improved accuracy of reaching 99% in prediction. The studies can be further extended to make performance analysis with the existing system.

## Figures and Tables

**Table 1:** UCF101 characteristics summary.

Actions	101
Slides	15320
Groups per act	50
Slides per group	5–8
Mean slide of distance	8.67 sec
Time duration	1200 mins
Min slide length	1.02 sec
Max slide length	61.43 sec
Frame frequency	30 fps
Resolutions	1320 × 1240
Video	Yes (100 actions)

**Table 2:** Result analysis of extracting features and continuing to learn in DL-DBN.

Extracting features and continuing to learn in DL-DBN							
Dataset	Feature extraction	Slides	Supervised/unsupervised feature learning	Resource	Time duration (mins)	Characteristics of DBN deducing model	
						Object recognition (sec)	Action recognition (sec)
UCF101	9	15320	Dynamic	TV, movies, you tube	1200	8.67	8.67

**Table 3:** Result Analysis of recognizing human sports behaviour using DL-DBN.

Recognizing human sports behaviour using DL-DBN			
	Supervised feature extraction	Unsupervised feature extraction	Soft max classification
Count	59.00	59.01	59.00
Mean	45.70	148.18	35.25
Std	4.77	5.29	3.94
Min	38.00	135.50	26.50
25%	49.50	147.04	17.56
50%	31.50	128.06	36.53
75%	35.00	151.03	39.51
Max	54.00	167.09	47.58

**Table 4:** Performance result analysis for sports spatiotemporal feature using DL-DBN.

Performance analysis for sports spatiotemporal feature using DL-DBN			
	DL-DBN	(Un)supervised feature extraction	Soft max classification
Count	59.76	49.01	52.87
Mean	45.51	128.45	29.75
Std	4.98	3.29	4.94
Min	38.45	125.58	21.58
25%	49.78	137.16	27.66
50%	31.41	118.27	46.43
75%	35.89	141.67	31.71
Max	54.63	117.52	39.68

**Table 5:** Result Analysis UCF101 dataset contains a classification models of various behaviours.

UCF101 dataset contains a classification models using DL-DBN										
Dataset	Feature extraction	Slides	Supervised/unsupervised feature learning	Resource	Time duration (mins)	Min slide length (sec)	Max slide length (sec)	Resolutions	Characteristics of DBN deducing model	
									Object recognition (sec)	Action recognition (sec)
UCF101	9	15320	Dynamic	TV, movie, you tube	1200	1.02	61.43	1320 × 1240	8.67	8.67

## Data Availability

The experimental data used to support the findings of this study are available from the corresponding author upon request.
